# Evaluation of assumed tumour volume in multiple myeloma using dual-energy spectral CT and its correlation between haematological findings

**DOI:** 10.1016/j.ejro.2025.100675

**Published:** 2025-07-29

**Authors:** Tetsuya Kosaka, Chisaki Masuda, Sachiho Tatebe, Risen Hirai, Akira Tanimura

**Affiliations:** a Department of Diagnostic Radiology, Tokyo-Kita Medical Center, 4-17-56 Akabanedai, Kita-ku, Tokyo, Japan; b Department of Haematology, Tokyo-Kita Medical Centre,4-17-56 Akabanedai, Kita-ku, Tokyo, Japan; c Department of Diagnostic Radiology, Kitasato University School of Medicine, 1-15-1 kitazato, Minami-ku, Sagamihara-shi, Kanagawa, Japan

**Keywords:** Multiple myeloma, Dual-energy spectral CT, Tumour volume, β2-microglobulin

## Abstract

**Objectives:**

To measure the assumed tumour volume in the humerus of patients with multiple myeloma using dual-energy spectral computed tomography (DESCT) and to evaluate the correlation with haematological indicators.

**Methods:**

We retrospectively analysed 82 DESCT examinations of 22 patients diagnosed with multiple myeloma. After extracting the bilateral humeri and removing the bone tissue, we measured the volume of the assumed tumour area using a single threshold based on Hounsfield unit values and double thresholds using material density images. We analysed the correlations between tumour volume and haematological indicators (β2-microglobulin, M-protein, free light chain, albumin, lactate dehydrogenase) and the trends after treatment intervention.

**Results:**

A moderate correlation was identified between the assumed tumour volume in the initial scan and the β2-microglobulin level, with a correlation coefficient of ρ = 0.69 for the volume calculated from a single threshold value of Hounsfield unit and ρ = 0.57 for the volume calculated from a double threshold value of the bone(fat) material density image. No significant correlation was found between the assumed tumour volume and the M-protein or free light chain levels. In patients who underwent three or more follow-up evaluations after the initial examination, there was a similarity in the changes in the assumed tumour volume and β2-microglobulin levels after treatment.

**Conclusion:**

Extracting assumed tumour volume using DESCT has sufficient potential as a biomarker for multiple myeloma.

## Highlights

• Extraction of assumed tumour volume of multiple myeloma using dual energy CT.
• Assumed tumour volume and β2 microglobulin level has a moderate correlation.
• Trends in assumed tumour volume and β2 microglobulin level after treatment are similar.
• Assumed tumour volume has sufficient potential as a biomarker for multiple myeloma.

## 1 Introduction

Multiple myeloma is a haematopoietic malignancy caused by malignant transformation of plasma cells. Multiple myeloma is classically characterized by multiple osteolytic lesions at diagnosis, and imaging plays an important role in the diagnosis of multiple myeloma. With recent advances in imaging technology, the current diagnostic criteria from the International Myeloma Working Group (IMWG) include the presence of one or more osteolytic lesions on computed tomography (CT) or positron emission tomography / computed tomography (PET/CT), and multiple focal lesions ≥ 5 mm on magnetic resonance imaging (MRI) as biomarkers of malignancy [1], [2].

Imaging is used not only for diagnosis, but also for follow-up and evaluation of treatment response [3], and many centres perform whole-body magnetic resonance imaging and CT. However, haematologic changes due to disease progression or treatment response are not always visually reflected in the images. CT is excellent at detecting osteolytic lesions and is useful for both diagnosis and evaluation at the time of progression, but even when the lesion responds to treatment, bone tissue regeneration is limited, so it may be difficult to evaluate treatment efficacy based on osteolysis alone. On the other hand, whole-body MRI has been reported to be difficult to differentiate between normal bone marrow and tumour invasion in approximately 30 % of multiple myeloma patients [4] and is superior for the assessment of progressive disease but inferior for evaluating complete response and objective response [5], and there are some signal changes that make it difficult to determine treatment response.

Therefore, if the tumour volume itself could be quantified, it might be a useful quantitative index to evaluate treatment efficacy. Although it would be desirable to be able to detect changes in tumour volume throughout the body as well as locally in order to assess progress by imaging [2], there are currently technical limitations.

However, because myeloma is thought to grow in patches throughout the body [6], capturing changes of tumour volume in some partial bone samples may provide useful information for progression monitoring and treatment efficacy evaluation.

In this study, bone-subtracted images of bilateral humeri were processed from dual-energy spectral CT (DESCT). We measured the volume of the assumed tumour area using a single threshold based on Hounsfield unit (HU) values and double thresholds using material density images. The correlation between the changes of assumed tumour volume and haematological indices and their trends after treatment intervention were investigated.

## 2 Methods

### 2.1 Patients

The study protocol was approved by our institutional ethics committee for retrospective evaluation of patient data with a waiver of informed consent (registration number: 395).

We retrospectively analysed whole-body CT examinations of patients with newly diagnosed multiple myeloma who visited our haematology department between December 2021 and January 2024. Of the 42 patients who were newly diagnosed with multiple myeloma during the observation period and received treatment, 25 patients who underwent at least two DESCT scans, one before and one after initial treatment, were included in the present study. However, the analysis was not possible for three of the 25 patients due to an error in the data storage process. Consequently, the analysis encompassed 82 DESCT examinations of 22 patients.

Measurements of β2-microglobulin, M-protein, free light chains (FLC), albumin, and lactate dehydrogenase were performed before the first treatment. After treatment, haematological data measured within two weeks of the DESCT scan were adopted.

### 2.2 CT-Protocol

All whole-body CT scans were performed on a 64-slice CT scanner (Revolution Frontier CT, GE Healthcare). Patients were positioned supine without elevating their upper extremities, and only the region from neck to pelvis was scanned using dual energy. No contrast was used. The following scanning and reconstruction parameters were used: collimation (64 × 0.625 mm), fast kV switching dual-energy scan with 80 and 140 kVp; setting noise index 13.5 with automatic tube current modulation (tube current 503.6 mA ± 123.6, range 275–640 mA); slice thickness, 5 mm; spacing, 5 mm; helical pitch, 1.375; table speed, 55–110 mm/s; rotation time, 0.5–0.7 sec/rotation; and adaptive statistical iterative reconstruction-V (ASiR-V, GE Healthcare), 30 % standard. For the purpose of visual evaluation, images with a slice thickness of 5 mm were utilised; however, all image data employed for analysis were obtained with a slice thickness of 0.625 mm or 1.25 mm.

### 2.3 Evaluation of image data

DESCT images were post-processed using Advantage Workstation 4.7 (GE Healthcare). As the appropriate threshold value for measuring assumed tumour volume was not established, the threshold value was provisionally determined as follows. From among patients classified as ISS stage III, one patient presenting gross bone resorption lesions and extramedullary lesions was selected as a model case. Multiple ROIs were then set in the solid tumour area of this patient. Each voxel density value was displayed using a GSI scatter plot (bone(fat) and bone(water)), and a radiologist visually determined a double threshold value that included approximately 80 % of the voxels as a provisional value. The HU threshold was provisionally set at 45–80 HU on the basis of our own empirical experience.

**Fig. 1:**
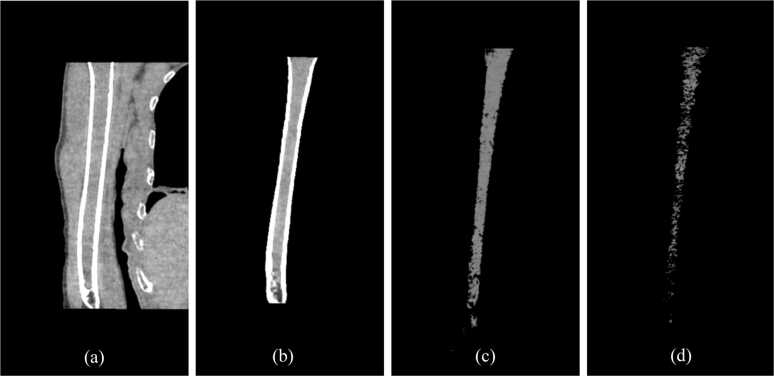
Example images of a 64-year-old female patient with multiple myeloma for visualisation and volume measurement of the assumed tumour. (a)–(b) The target area, extending from the surgical neck of the humerus to just above the olecranon fossa, was extracted from DESCT images. (c) Utilising a single threshold of 45–80 HU, the assumed tumour area was left, and its volume was measured. Using a single threshold (45–80 HU), the volume is measured, leaving only the assumed tumour area. (d) Utilising a double threshold (bone (60–130 mg/cm^3^)–fat (925–1000 mg/cm^3^)), the assumed tumour area was left, and its volume was measured. DESCT, Dual-Energy Spectral Computed Tomography; HU, Hounsfield Unit.

From all DESCT scans, images from the surgical neck of humerus to just above the olecranon fossa were extracted (Figs. 1(a), 1(b)). A radiologist with over 15 years of experience determined the optimal site for humeral extraction based on anatomical landmarks (surgical neck and olecranon fossa) identified on CT images. The results of this slice position were then recorded. In the longitudinal evaluation of the same patient, the humeral bone volume extracted in accordance with anatomical indicators was refined so that the difference from the initial measurement was within ±1 %.

Then we measured the assumed tumour volume using the single threshold of HU values (Fig. 1(c)). Next, we converted to two different material density images (bone(fat) and bone(water)) using the double thresholds. Fig. 1(d) shows the material density image of bone (fat) using the double threshold. We measured the assumed tumour volume using these thresholds.

### 2.4 statistical analyses

All statistical analyses were performed using R: A Language and Environment for Statistical Computing version 4.3.2 R Foundation for Statistical Computing, Vienna, Austria) and RStudio: Integrated Development Environment for R version 2024.04.2.764 (Posit Software, PBC, Boston, MA).

The distribution of assumed tumour volume and haematological indicators in the initial examination did not follow a normal distribution, as determined by the Shapiro–Wilk test. Spearman's correlation coefficient was thus calculated.

Statistical analysis was then performed on the differences between assumed tumour volumes calculated from bone(fat) thresholds in the three groups of the ISS classification. In this statistical analysis, given that the Shapiro–Wilk test did not indicate normality, the Kruskal–Wallis test was followed by the Dunn–Bonferroni post-hoc test.

## 3 Results

A total of 22 patients met the inclusion criteria. Patients’ age at the initial examination ranged from 51 to 87 years, with a median age of 65 years. The international staging system (ISS) and revised ISS stages were determined based on data from the initial examination, followed by CT scan. The number of days between the date of acquisition of haematological indicators and CT scan was limited to 14 or less, and the data were used as the target for analysis. 94 % of the haematological data were taken within 1 week of the CT scan. Patient characteristics are shown in Table 1.

**Table 1:** Demographic and baseline characteristics.

	Overall
Characteristics	n = 22
Age (years), median (IQR)	65 (58.5–72.5)
Sex	
Males (%)	12 (54.5)
Females (%)	10 (45.5)
Myeloma subtype	
IgG (%)	10 (45.5)
IgA (%)	7 (31.8)
BJP (%)	5 (22.7)
International Staging System	
Stage I (%)	5 (22.7)
Stage II (%)	8 (36.4)
Stage III (%)	9 (40.9)
Revised International Staging System*	
Stage I (%)	2 (9.1)
Stage II (%)	13 (59.1)
Stage III (%)	6 (27.3)

Abbreviations: IQR, interquartile range; BJP, Bence Jones protein

* One person did not meet the inspection requirements

A total of 82 CT scans were performed, and the volume of assumed tumour was calculated for each target area. The volume of target humeral bones ranged from 128.05 to 287.88 cm^3^, with a median of 193.84 cm^3^. The volume of the assumed tumour area, calculated from the bone (fat) threshold ranged from 0.66 to 12.13 cm^3^, with a median of 1.82 cm^3^. (Table 2).

**Table 2:** Measurement results (Initial examination).

	total = 22	median (IQR)
serum β2 microglobulin (mg/L)		4.05 (2.9–8.38)
Monoclonal protein (mg/dL)		
IgA	n = 7	2999 (2122–4407)
IgG	n = 10	4887 (3828–8339)
BJP (urine)	n = 5	9865 (4026–11306)
involved Free light chain (mg/dL)		
IgA-κ	n = 3	950.7 (488.2–1001)
IgA-λ	n = 4	1876 (231.8–3917)
IgG-κ	n = 7	356.4 (60.1–1038)
IgG-λ	n = 3	605.6 (450.0–607.6)
BJP-κ	n = 4	7666 (3029–11306)
BJP-λ	n = 1	9865.2
LDH (U/L)		162 (131–209)
Albumin (g/dL)		3.5 (2.9–3.9)
cCa (mg/dL)		10.2 (9.5–10.6)
Creatinine (mg/dL)		0.82 (0.64–1.07)
Extracted humeral volume (cm^3^)		193.8 (172.3–223.2)
Assumed tumour volume		
double thresholding with bone(fat) (cm^3^)		1.82 (1.24–4.09)
double thresholding with bone(water) (cm^3^)		12.4 (6.40–15.6)
single thresholding with 45–80HU (cm^3^)		6.39 (4.65–9.58)

Abbreviations: IQR, interquartile range; BJP, Bence Jones protein; LDH, lactate dehydrogenase; cCa, corrected Calcium

**Fig. 2:**
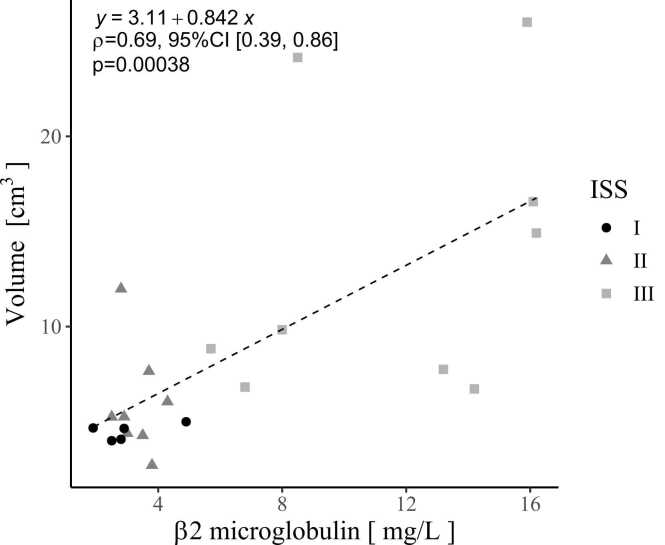
The Spearman's correlation coefficient between the volume of an assumed tumour measured using a single threshold (45–80 HU) and β2-microglobulin levels was positively moderate correlated. ρ = 0.69 [95 % CI 0.39–0.86], p < 0.01. HU, Hounsfield Unit; CI, Confidence Interval.

**Fig. 3:**
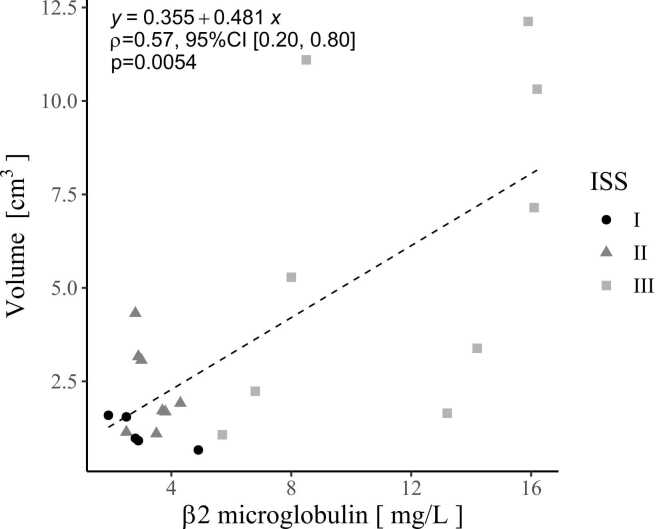
The Spearman's correlation coefficient between the volume of the assumed tumour measured using a double threshold (bone (60–130 mg/cm^3^)–fat (925–1000 mg/cm^3^)) and β2-microglobulin levels was positively moderate correlated. ρ = 0.57 [95 % CI 0.20–0.80], p < 0.01. CI, Confidence Interval.

The estimated tumour volume, calculated from the HU value or bone (fat) threshold measured at the initial examination, and the β2-microglobulin value demonstrated a significant correlation (p < 0.01) when Spearman's correlation coefficient was calculated. The correlation coefficients were ρ = 0.69 [95 % CI 0.39–0.86] and 0.57 [95 % CI 0.20–0.80], respectively, indicating a moderate correlation (Fig. 2, Fig. 3). The assumed tumour volumes, which were calculated from the bone(water) threshold, and β2-microglobulin levels did not show a significant correlation (p = 0.1). There was no significant correlation between any assumed tumour volume and other haematological indicators (M-protein, FLC, albumin, lactate dehydrogenase).

**Fig. 4:**
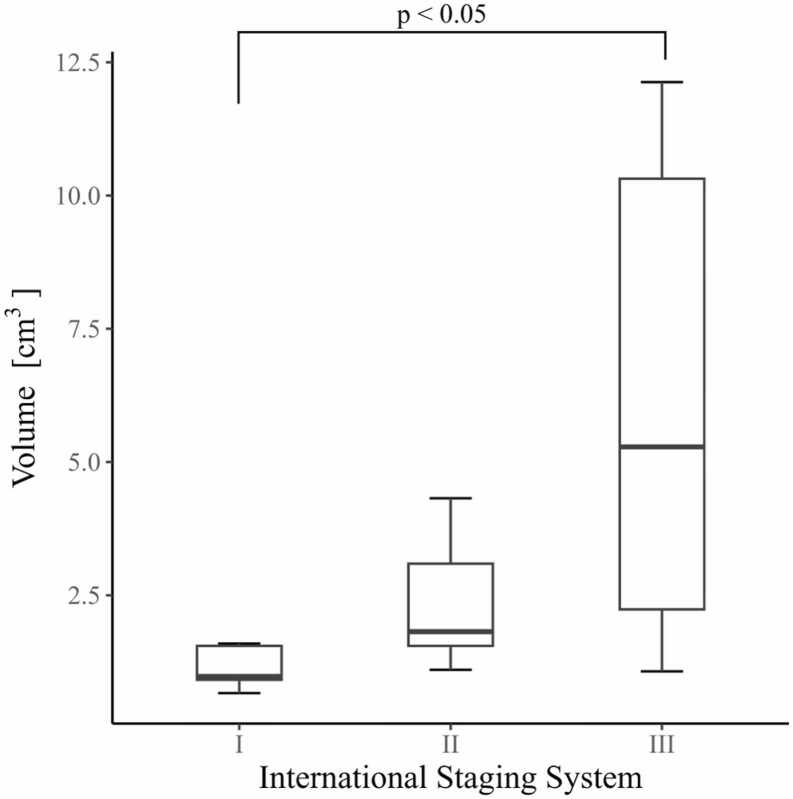
Box-and-whisker plot of the assumed tumour volume for each ISS stage. A significant difference was observed between stage Ⅰ and Ⅲ at (p < 0.05). Volume, Volume of the assumed tumour using a double threshold (bone (60–130 mg/cm^3^)–fat (925–1000 mg/cm^3^)); ISS, International Staging System.

A comparative analysis was conducted to ascertain the disparities in estimated tumour volumes derived from the bone (fat) threshold across the three groups classified according to the ISS (International System of Staging) classification. The p-values between each group were p = 0.0853 between Ⅰ and Ⅱ, p = 0.0021 between Ⅰ and Ⅲ, and p = 0.2269 between Ⅱ and Ⅲ. A significant difference was observed between Ⅰ and Ⅲ at p < 0.05 (Fig. 4).

**Fig. 5:**
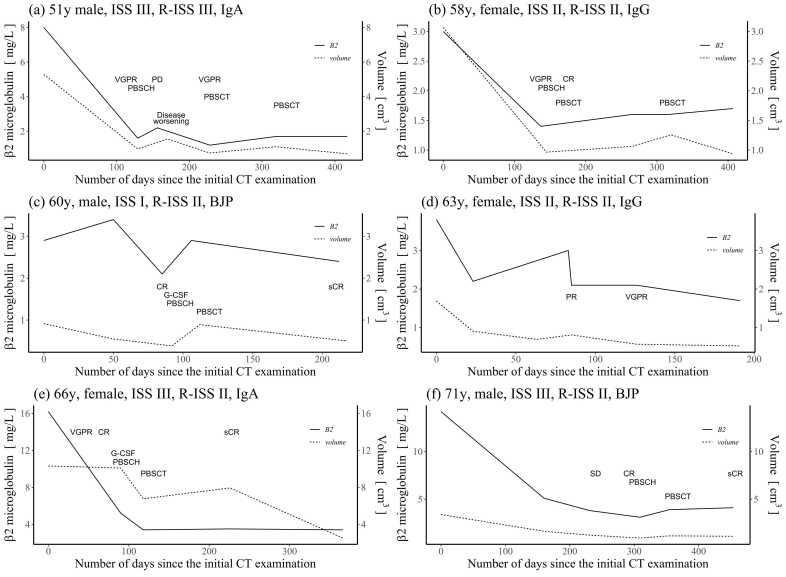
(a)–(f): Trends in β2-microglobulin and assumed tumour volume since the first examination. Patients who have been tested more than five times are shown as representative examples. The horizontal axis shows the number of days since the first examination. The patient characteristics displayed are in the order of age, gender, ISS stage, R-ISS stage, and type of myeloma. The assumed tumour volume exhibited fluctuations in accordance with the treatment effect and disease progression. Additionally, fluctuations were observed subsequent to the administration of G-CSF in isolation or following the harvesting and transplantation of haematopoietic stem cells. CR, Complete Response; G-CSF, Granulocyte Colony Stimulating Factor; ISS, International Staging System; PBSCH, Peripheral Blood Stem Cell Harvest; PBSCT, Peripheral Blood Stem Cells Transplantation; PD, Progressive Disease; PR, Partial Response; sCR, stringent CR; SD, Stable Disease; VGPR, Very Good Partial Response.

The results of patients who underwent three or more tests after the initial examination showed similarities in changes of assumed tumour volume calculated from the threshold of bone(fat), and β2-microglobulin values after treatment. Trends of 6 patients who underwent 5 or more examinations are shown in Fig. 5(a)–(f) as representative examples. Other than treatment effects and disease progression that cause fluctuations, the assumed tumour volume also changed after granulocyte colony stimulating factor (G-CSF) administration and stem cell transplantation. However, the cause has not yet been determined. With regard to β2-microglobulin levels, there were a few cases of elevations that deviated from the anticipated treatment effect, yet there were no renal function deterioration, and the underlying mechanism remained elusive. The fact that M-protein and FLC values demonstrated a trend of reduction after treatment corresponded to the trends of assumed tumour volume and β2-microglobulin levels, but the timing of reduction and the trends after reduction did not show homology.

## 4 Discussion

This study demonstrates that, despite the humerus is only a partial element, it is feasible to extract and quantify the volume of tumour-assumed tissue using DESCT. We found that this approach had the potential to evaluate the tumour burden at diagnosis and the effect and course of treatment using the assumed tumour volume.

Multiple myeloma is a disorder of plasma cells that causes bone marrow infiltration of clonal plasma cells and osteolytic lesions, so diagnosis and evaluation require a systemic search for intramedullary and extramedullary lesions. CT is particularly effective in detecting osteolytic lesions and is also often used to assess extramedullary lesions and foci of infection. According to the consensus of the IMWG, whole-body CT is the first choice for initial evaluation of multiple myeloma. Although not defined, it is also often used for follow-up evaluation after treatment [1], [2], [7].

However, since multiple myeloma does not always cause bone lesions homogeneously throughout the body, and it is thought that 60 % of patients do not have osteolysis [2], there is a limitation in sensitivity for detecting non-lytic myeloma infiltration. Especially, when the tumour volume is small and the ISS stage is low, it is difficult to visually detect the presence of tumour or the increase or decrease in the tumour as it progresses or improves.

The number of focal lesions has been shown to correlate with prognosis, suggesting that tumour volume itself is a prognostic factor. [8], [9]. However, estimating the amount of viable tumour in the bone accurately remains an unsolved and important issue.

In this study, which only evaluated the humerus, a moderate correlation was suggested between the tumour volume estimated using the single threshold of HU values or the double threshold of bone(fat) and the biomarker β2-microglobulin. Elevation of serum β2-microglobulin levels may reflect the amount of tumour in patients with haematologic malignancies, particularly multiple myeloma, and have been shown to be a very effective and independent indicator of survival in multiple myeloma [10], [11].

Previous studies have reported that the detection of multiple myeloma lesions in long bones (nodular or diffuse) is a prognostic factor [12], and that changes in these lesions correlate with treatment response [13]. The method in this study allows for the confirmation of minute lesions as numerical values, even if lesions cannot be visually detected.

At present, it is said that MRI, which is more sensitive to invasive disease, is more suitable than standard CT for evaluating bone marrow infiltration in multiple myeloma, and it is reported that MRI can provide markers of tumour burden in patterns of normal/diffuse infiltration [7], [9]. On the other hand, there are also numerous reports that suggest dual-energy CT may also be a promising approach for evaluating invasive lesions [14], [15], [16], [17]. In the bone marrow that has been diffusely infiltrated by tumour cells, it is thought that the high degree of disappearance of fat cells and the increase in infiltration by tumour cells have significant effect on the HU value of the lesion site [16], [18], and this may be the reason why DESCT using bone(fat) had a better correlation with β2-microglobulin levels than bone(water) in this study.

There was no significant correlation between assumed tumour volume and M-protein or FLC. This may be due to the fact that the production of M-protein and FLC is greatly influenced not only by the amount of tumour but also by the nature of the tumour (e.g. secreted amount). Furthermore, as the stage of the disease progresses, the distribution of the tumour becomes more biased, and it is difficult to obtain results representatively from a single site such as the humerus. In this study, the distribution of assumed tumour volume in ISS stage Ⅲ varied widely, which may reflect a significant difference in disease progression between patients of stage Ⅲ and a great bias in tumour growth in various parts of the body (e.g. a substantial tumour volume in the entire body but a relatively small volume in the humerus).

The change of assumed tumour volume after treatment intervention was also followed in this study. Trends in β2-microglobulin levels and assumed tumour volume calculated using thresholds of HU values or bone(fat) showed homology.

As a result of treatment, the contents of bone resorption lesions that have attenuation values comparable to those of muscle show significant reduction of HU values and become equivalent to those of water or fat [7]. In a study of dual-energy CT after radiotherapy for multiple myeloma, fatty changes and cell loss were reported to be more prevalent than lesion stiffening [19], which suggests that the double threshold of bone(fat) may be more useful in evaluating the progress of the disease.

We consider that the use of the double threshold of bone(fat) has higher potential for evaluating treatment efficacy and disease progression than the single threshold of HU values. However, in this study, correlation between the volume based on the bone(fat) threshold and the β2-microglobulin level was almost the same as the correlation between the volume based on the HU threshold and the β2-microglobulin level.

Treatment efficacy is evaluated primarily through haematological indicators (M-protein, FLC, etc.). But in cases such as low-secretory and non-secretory myeloma, haematological evaluation has limitations in assessing myeloma lesions. For this reason, imaging evaluation is used as a diagnostic aid, and we believe that our method will also be of assistance.

However, among other transitions due to therapeutic intervention, a slight increase in tumour volume was observed after G-CSF administration and during at the post-transplant stage. Although the cause has not yet been identified, there are reports that reconversion due to haematopoietic stimulation and anaemia impair the accuracy of treatment evaluation [7]. G-CSF exerts its influence on bone marrow and is involved in the increase of myeloid cells and the mobilisation of haematopoietic stem cells [20], [21]. However, the main biological effect of G-CSF is to promote the proliferation and differentiation of neutrophils from committed progenitor cells [22]. This phenomenon may contribute to the increase in bone marrow cells, but the extent of its influence is unknown. Peripheral blood stem cell transplantation is a procedure that is performed following bone marrow destruction therapy [20]. Consequently, a reduction in bone marrow cells, including tumour cells, prior to transplantation and an increase in normal bone marrow cells after transplantation are expected. While these may potentially impact assumed tumour volume fluctuations, the absence of pathological evidence to corroborate this hypothesis remains a limitation. This indicates that visually assessing remaining viable lesions becomes even more difficult at certain times after treatment.

In regard to the quantitative evaluation of multiple myeloma, there have been attempts to calculate tumour volume by PET/CT and to evaluate tumour volume by diffusion limited zone quantification [23], [24], but to the best of our knowledge, there are no studies that have evaluated tumour volume by DESCT. So far, visual evaluation has been mainly based on MRI patterns and HU values in CT, and quantitative data extraction of the tumour from the images has not been sufficient. Renyang Gu et al. have successfully used their original program to isolate bone from whole body and analyse resorption values [17], and the quantification of tumour volume from bone of the whole body may become possible due to these efforts.

There are several limitations to this study. One clear limitation is that a whole-body bone assessment was not accomplished, which is an issue for the future. In light of the general-purpose tools currently at our disposal, it is challenging to analyse the assumed tumour due to the inability to remove the intervertebral spaces and joint cavities when extracting and analysing the vertebrae and pelvic bones. Second, the sample size was relatively small, and the timings of the examinations were not consistent due to the retrospective nature of the study. Third, there was not always pathological evidence to support the thresholds we used to calculate the assumed tumour volume, and the optimal thresholds were not fully investigated. Furthermore, verification of this virtual tumour volume is limited due to the lack of histological confirmation. Fourth, this study used β2-microglobulin as the reference standard for assumed tumour volume estimation. However, it should be noted that this does not necessarily reflect the actual tumour volume. Fifth, the utilisation of high-resolution bone algorithm imaging technology is expected to improve measurement accuracy. Nevertheless, concerns regarding noise and increased radiation exposure have prevented its introduction at our facility without sufficient evaluation.

In conclusion, this study shows that extracting assumed tumour volume using DESCT has sufficient potential as a biomarker for multiple myeloma. We assume that even a partial evaluation, such as the one presented in this study, can be representative at an early disease stage. In the future, we consider that it is necessary and possible to specifically estimate the systemic tumour burden as a volume value.

## Funding Statement

The present study did not receive any specific grant from funding agencies in the public, commercial, or not-for-profit sectors.

## Funding

None.

## Ethical Statement

The present study was conducted in accordance with all the relevant national regulations, institutional policies and the tenets of the Helsinki Declaration. Ethical approval for this study was obtained from our institutional ethics committee (registration number: 395). The requirement for written informed consent from patients was waived due to the retrospective nature of the study.

## CRediT authorship contribution statement

**Akira Tanimura:** Resources. **Tetsuya Kosaka:** Writing – original draft, Visualization, Validation, Supervision, Methodology, Formal analysis, Conceptualization. **Chisaki Masuda:** Investigation, Data curation. **Sachiho Tatebe:** Writing – review & editing. **Risen Hirai:** Writing – review & editing, Resources, Investigation.

## Declaration of Competing Interest

The authors declare that they have no known competing financial interests or personal relationships that could have appeared to influence the work reported in this paper.

## Data Availability

Data generated or analysed during the study are available from the corresponding author by request.
